# Decreasing Shear Stresses of the Solder Joints for Mechanical and Thermal Loads by Topological Optimization

**DOI:** 10.3390/ma13081862

**Published:** 2020-04-15

**Authors:** Jan Awrejcewicz, Sergey P. Pavlov, Anton V. Krysko, Maxim V. Zhigalov, Kseniya S. Bodyagina, Vadim A. Krysko

**Affiliations:** 1 Department of Automation, Biomechanics and Mechatronics, Lodz University of Technology, 1/15 Stefanowskiego Str., 90-924 Łódź, Poland; 2 Department of Mathematics and Modelling, Saratov State Technical University, Politehnicheskaya, 77, Saratov 410054, Russian; pspsar@yandex.ru (S.P.P.); zhigalovm@ya.ru (M.V.Z.); bodksen@mail.ru (K.S.B.); tak@san.ru (V.A.K.); 3 Department of Applied Mathematics and Systems Analysis, Saratov State Technical University, Politehnicheskaya 77, Saratov 410054, Russian; antonkrysko@gmail.com

**Keywords:** layered structures, solder, adhesion, thermomechanics, numerical analysis

## Abstract

A methodology for obtaining the optimal structure and distribution for the gradient properties of a material in order to reduce the stress level in a soldered joint was constructed. The developed methodology was based on a combination of topological optimization methods (the moving asymptotes method) and the finite elements method; it was first implemented to solve problems of optimizing soldered joints. Using the proposed methodology, a number of problems were solved, allowing one to obtain optimal structural characteristics, in which a decrease in stress is revealed. Designing compounds using this technique will provide more robust designs. The proposed technique can be applied to a wide class of practical problems.

## 1. Introduction

Most structures in the aviation, shipbuilding and rocket industries are aimed at achieving a high resistance against various loads while being simultaneously lightweight. When designing and manufacturing mechanical (or electromechanical) structures, the choice of bonding components plays a crucial role in achieving the industry-required characteristics. Mechanical bonding, including beating and twisting, is widely used in design, although a technology of adhesion joints either treated separately or matched with mechanical fastening can essentially improve mechanical characteristics from the point of view of stiffness, strength and durability [1,2,3]. Solder and adhesive joints stand are frequently used advantageous ways of connecting different materials. 

Solder and adhesive joints have advantages over other alternative ways of fastening due to stress distribution over a wider area than that based on adhesive joints or beating fastening. In contrast to the non-uniform load distribution under fastening by bonding elements, the load transmission through solder or adhesive components is continuous along the entire connecting layer. It enables the production of simple and lightweight joints. In other words, such a joint makes it possible to decrease the weight of the structure while maintaining the required mechanical loading capacity. That is why solder and adhesive joints are so often used in the design of mechanical systems [4,5,6,7,8]. 

Although it is well known that adhesive is one of the best methods for fastening composites with metals, the non-uniform distributions of stress/strain generated in bond-lines under shear loads often cause damage to bonded joints. However, the solder joints, suffer from temperature changes in the exploitation process due to the different linear temperature expansion coefficients of the bonded materials.

Most failures of solder joints that occur in electronic systems are problems caused by thermal mismatch between various materials, including those involved in the creation of electronic systems. During manufacture and operation, the structure goes through various temperature cycles that cause thermal expansion. Materials cannot expand freely because they are limited by packaged assembly. Therefore, significant stresses arise in soldered joints. In [9], these stresses causing the destruction of soldered joints were mathematically modeled using software and a comparison was made with experimental results. The thermomechanical reliability of the solder connection has been extensively investigated using finite element method [10,11].

Thermal stresses generated by solder are changed in wide intervals depending on the nature of the temperature fields, configurations and material properties. High stresses can cause cracks, thermoplastic deformations and other harmful effects which reduce the load carrying ability of the skip solders. It is expected that the optimization of the solder layer topology can eliminate problems associated with the occurrence of peak stresses in the intervals of the imposed structure and technological constraints. In the case of adhesive joints composed of functionally graded material/composite, stress fields are also often affected by the lack of symmetry. The maximum stress–strain characteristics are near the ends of the joints and suggest their rupture [12].

Groth and Nordlund [13] proposed the optimization of the shape of bonded joints to create strong and light joints under static loading by introducing optimal profiling of adherents. A significant decrease in the level of stress of the adhesive layer was obtained considering single/double laps and a double strap.

Hildebrand [14] analyzed single lap joints between fiber-reinforced plastics and metals using the non-linear finite element methods. Different shapes of adhesive fillet, rounded edges, reverse tapering and denting were used to increase the joint strength.

Rispeler et al. [15] employed the evolutionary structural optimization approach in order to optimize the shape of adhesive fillets in tabs of tensile test specimens. They showed a reduction in the maximum principal stresses in the adhesive for all considered cases. 

Taib et al. [16] described the effects of joint configuration, defects, humidity, adhesive stiffness of glass-fiber-reinforced vinyl-ester composite laminates. In particular, they demonstrated a decrease of failure load and displacement associated with the increase of the adhesive layer thickness and aging of the joint in a hot and humid environment.

Hints and design methods to increase the load capacity of various joints and the state-of-the-art approaches aiming at the modification of the geometry of adhesive joints as well as their influence on the magnitude of stresses and effective bonding forces were considered in [3]. The properties of adhesive material, the thickness of adhesive layer, length and width of the adhesive contact layer as well as residual stresses were taken into account. 

Silva and Adams [17] combined two adhesives (one for strength at high temperatures and one for strength at low temperatures) to design joints required for the fuselage of a supersonic aircraft. A numerical analysis based on finite element models made it possible to reduce the stress distribution and design the best titanium/titanium and titanium/composite double lap joints.

Haghani et al. [18] developed a stress reduction method to modify the geometry of the adhesive joint by either tapering the laminate end or by adding an adhesive fillet. An optical measurement system was used to monitor the strain field. It was shown that normal tapering of the laminate did not affect the shear and principal stress components.

Lang and Malick [19] investigated how the geometry of adhesive spew affects peak stresses and stress distribution on adhesively bonded single lap joints. A linear 2D strain analysis was carried out using the ANSYS finite element software. It was illustrated how by shaping the spew, a smoother transition in joint geometry reduced the stress concentration at the substrate–adhesive interface.

There is a long list of investigations that focused on the parametric optimization of adhesive lap joints by using appropriate change of spew and chamfer angles [16,20,21,22,23,24,25]. Although the authors claimed that the combination of the latter two characteristics allowed them to achieve bonding in adhesive lap joints with the highest strength, the given values are not clearly defined. 

Sancaktar and Simmons [26] carried out a parametric study using finite element analysis for three different model adhesives: rubber toughened film epoxy, a styrene-butadiene-styrene block copolymer, and a metal filled brittle epoxy adhesive. The experiments show that the increase in the joint strength with the introduction of notches which was in agreement with the finite element analysis.

Despite the parametric optimization carried out, non-parametric optimization, including topological optimization and optimization of the form, can be used to determine the strength of the adhesive joint.

Algorithms based on non-parametric optimization are aimed at structural optimization without taking into account the a priori chosen variables. Therefore, the basic advantage of the non-parametric optimization lies in defining the best form/topology without any a priori knowledge of the final construction design. However, the use of non-parametric approaches to the geometry of the adhesive or solder joint is not widely known and is treated rather marginally in the available literature.

The first attempt to optimize adhesive joints using the adhesive variable was described in [13]. The work was focused on achieving joints with maximum light in the static conditions by changing the profiles of adhesives. It was shown how the optimization of the form causes a significant reduction of stresses in the adhesive layer. The evolutionary method of structural optimization was applied to optimize the form of the adhesive layer in order to achieve minimum stresses [15].

Kaye and Heller [27] developed an automated sensitivity-based optimization procedure for the optimal design of free-form bonded repairs and lap joints to reduce adhesive stresses. Significant improvements were presented in relation to conventional designs.

Ejaz et al. [28] studied the applicability of non-parametric structural optimization algorithms for the optimization of adhesive joints of the types: single lap, double lap and double lap strap. Stress reduction in the adhesive layers was achieved. 

In [29], ANSYS software was used to analyze the structure effect and shape of a soldered joint on fatigue life due to elastoplastic deformation of the electronic package; these factors influenced strongly the structure and shape of the solder.

Tian and Wang [30] proposed schemes for analyzing the shape and reliability of a soldered joint. By changing the pad’s size, the shapes of soldered joints with different volumes of solder were predicted. Using the finite element method, the characteristics of the stresses and strains distribution in soldered joints under thermal load were analyzed.

In article [31], the shape’s and height’s influence of the soldered joint on the thermal load time was studied. Experimental data indicate that the solder shape stands for the dominant factor affecting the crack initiation time.

In [32], the shape and location optimization of soldered joints under vibration and shock conditions was studied in order to increase their reliability and durability. Based on the experiment, a finite element model was constructed that is close to the assembly model. Then, the modified genetic algorithm (global optimization) was used to determine the shape and location of soldered joints under shock loads. The optimum results showed that a solder joint with an optimal arrangement and shape had a lower maximum deformation, which increased the reliability of the solder joint.

In general, the optimization was focused on either the form of neighborhood elements or the form and position of the adhesive layers. The alternative strategy includes introduction of the variation/graduation of the properties along the thickness or length of the adhesive layers. It includes the modification of material properties or the geometry of adhesives which are called the functionally graded adhesive joints. In order to achieve the required properties of the adhesive, various approaches were introduced: (i) softening the brittle adhesive with rubber; (ii) supplementing additional stiffness of the flexible adhesive with glass microspheres or silica nanoparticles [24,25]; (iii) mixing of various bonded adhesives and control of the polarization level by induction heating [26,27,28]. Other variants of possible joint modifications were described in [30].

In general, the optimization was focused on either the form of neighborhood elements or the form and position of the adhesive or solder layers. One of the approaches indicates the use of a softer adhesive at the boundary ends and a more rigid one in the joint center. The second concept presents the contraction of the adhesive at the joint ends which allows for preserving neutral stresses and decreasing peak stresses in the adhesive [32,33]. 

In recent years, a series of works devoted to the study of adhesive properties and occurring stresses have been published [34,35,36]. The studies were carried out on the basis of analytical approaches [37,38], numerical modeling [39,40] as well as a combination of both techniques [41,42,43]. 

Studies based on continuous change of properties use a linearly elastic material model [35,36].

Independently of the approach for getting gradient properties of the adhesive layer, a control of the production process was employed to obtain the required optimized distribution/change of material properties. Owing to the complexity of the problem, many researchers began to reduce stress/deformation by using bi-adhesive with different stiffnesses of layers, allowing for easier control [36,41].

Brebia and Kassab [44] combined many papers with recent information regarding computer-aided structural design optimization software and integrated packages for structural optimization. The presented subjects included optimal control, shape optimization, linear and non-linear structural optimization, component reliability and optimal design of reinforced members.

París et al. [45] presented a strategy to deal with topology optimization based on minimum weight with stress constraints for topology optimization of continuum structures. The stiffness of the resulting structure was maximized for a given load case.

Qiu and Li [46] derived the KS (Keisselmeier-Steinhauser) global constraint function to reduce the members of stress constraints.

Stolpe and Svanberg [47] considered the discretized zero-one continuum topology optimization to find the optimal distribution of two linearly elastic materials in order to achieve the compliance minimum. They used a material interpolation model based on a certain rational function parameterized by a positive scalar such that the compliance was a convex function.

Le et al. [48] implemented an effective algorithm to resolve the stress-constrained topology optimization task. The applied technique combined the density filter for length scale control, solid isotropic material with penalization, and a new definition of stress to remove the singularity of stress and control local stress levels.

On the basis of the state of the art, it can be stated that even if non-parametric optimization was used, the existing optimization in the mentioned works and outside them concerned either the form of the bonded elements or the form and location of the adhesive layer. 

The overall solder joint reliability was estimated by the combination of operating conditions and system design. The working environment determined the temperature extremes that the structure must withstand as well as the possibility of mechanical stress. The above studies show that under the influence of temperature and mechanical loads, structural failure occurs due to peak tangential stresses near the edges of the joint. Therefore, the present work focuses specifically on reducing shear stresses.

To the best of our knowledge, methods of topological optimization to define the optimal structure of the solder or adhesive layers in the presence of thermoelasticity were not used in the existing literature.

Despite the complexity of manufacturing topologically optimal connection layers, the structure type optimal for obtaining the maximum bond strength for a specific design is of great interest, since it determines the target solution that must be achieved.

In this paper, a mathematical model is proposed and a methodology was developed for solving a solder class of thermoelastic problems of topological optimization with the aim to obtain the optimal structure and required gradient properties of the solder layer to decrease the level of shear stresses generated in it.

Solder joint failures are a common failure mode observed in electronic packages [49]. A number of problems were considered to reduce the shear stresses of joints with silver solder in thermoelastic three-layer packets subjected to mechanical and thermal loads by topological optimization.

## 2. Problem Statement

Thermal stresses are one of the most important factors in the production of Micro Electro Mechanical Systems (MEMS), and have negative consequences due to the occurrence of harmful cracks. They also reduce the length of their exploitation with the required functional properties. The difference in the linear heat transfer coefficients associated with different materials of the package implies the occurrence of both thermal stresses and deformations. Particular attention should be paid to solder between package layers that guarantee electrical contact between the MEMS electronic components. The change in temperature within maximum and minimum values has many negative effects, including the occurrence of stoppages in MEMS. 

**Figure 1:**
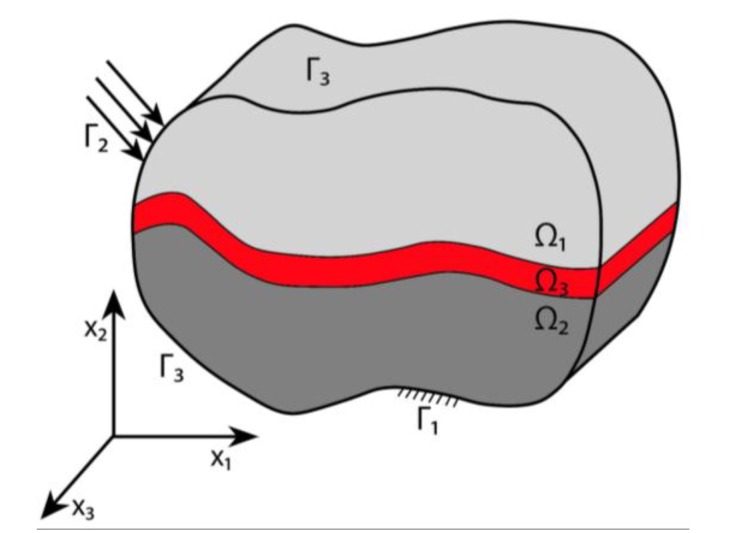
Computational model of a three-layer mechanical package.

In the following section, we present, due to its simplicity, a plane problem of thermoelasticity. We consider a package composed of an elastic non-homogenous body Ω exhibiting phase stress/strain state with regard to the x axis and consisting of three parts, as shown schematically in Figure 1 (parts Ω*_i_* are coupled and index *i* denotes the part number (*i* = 1,2,3)). The upper layer 1 stands for the foundation, the central layer 3 stands for the solder layer, while the bottom layer 2 plays the role of the base. The whole package is embedded into the temperature field *T*(**x**), **x** = {*x*_1_, *x*_2_}. We denote the temperature change *θ*(**x**) = *T*(**x**) – *T*_0_ with respect to its initial value *T*_0_. The Young modulus and the temperature coefficient of the linear extension in Ω*_i_* are denoted by *E_i_*(**x**) and *α_i_*(**x**), respectively. The 2D elastic part of Ω is bounded by the closed surface $\Gamma =\Gamma_{1}\cup \Gamma_{2}\cup \Gamma_{3}.$ It is assumed that we consider linear, elastic and isotropic material.

The boundary conditions are shown in Figure 1. Boundary Γ_1_ refers to the rigid clamping, boundary Γ_2_ is under the load of intensity **t**, whereas boundary Γ_3_ is free. The field of displacement (*u*_1_, *u*_2_) implies the following equilibrium equation:

(1) $$\sigma_{ij,\;j}=0\;\mathrm{in}\;\Omega ,\;\;\;\left(i,\;j\;=1,2\right),$$

where *σ_ij_* is the stress tensor. Linear deformations and displacements are coupled through the following relations

(2) $$\varepsilon_{ij}=\frac{1}{2}\left(u_{i,\;j}+u_{j,\;i}\right),\;\;\;i,\;j\;=1,2.$$

The stress–strain relations, in the case of plane stress state, are estimated via the Duhamel-Neumann principle

(3) $$\sigma_{ij}=E(x)(\varepsilon_{ij}-\alpha \left(x\right)\;\theta \left(x\right)\;\delta_{ij}),$$

(4) $$\tilde{E}\left(x\right)=E\left(x\right)/\left(1-\nu^{2}\right)$$

(5) $$\tilde{\alpha }\left(x\right)=\alpha \left(x\right)\left(1+\nu \right)$$

where *δ_ij_* is the Kronecker’s delta, *E*(**x**) is the Young modulus and *α*(**x**) is the linear coefficient of temperature expansion. In the case of a plane deformable state, the following substitution should be made: *E*(**x**) on the $\tilde{E}\left(x\right)$ by relation (4) and *α*(**x**) on the $\tilde{\alpha }\left(x\right)$ by relation (5), where ν is the Poisson’s ratio. Observe that the fields of displacement and temperature are coupled through Equation (3).

## 3. Formulation of Topological Optimization Problem

Analysis of vibrations in the solder joints shows that small solder thickness means the occurrence of shear stresses. They are concentrated at the ends of the solder and they achieve minimum at the solder center. This is why the goal of optimization is to decrease the peak values of stresses $\sigma_{12}$ in the solder layer. In fact, the optimization of the solder structure is focused on searching for the best solder distribution along Ω_3_ in order to obtain the minimum peak values of stresses $\sigma_{12}.$

Since a small number of constraints in the optimization problem plays a crucial role in computational processes, we start with only one constraint introduced on maximum stresses

(6) $$g(r)=\underset{\Omega_{3}}{\max}\left(\sigma_{12}(r)\right)\leq \bar{\sigma },$$

where r(x) is the construction variable and $\bar{\sigma }$ is the given value of the maximum shear stress in the solder. However, the maximum operators are not differentiable, which does not allow us to derive analytical sensitivity formulas under the occurrence of Equation (6). In order to smooth the mentioned operators, continuous functions matching local stresses to one global constraint were introduced [45,46,50].

In particular, París et al. [45] proposed the following global aggregation functions:

(7) $$g_{1}\left(r\right)=\frac{1}{\mu }\ln\left(\iint_{\Omega_{3}}e^{\mu \frac{\sigma_{12}\left(r\right)-\bar{\sigma }}{\bar{\sigma }}}d\Omega \right),$$

where *μ* is the penalty parameter having usually large values. Then, inequality (6) can be recast to the following form:

(8) $$g_{1}\left(r\right)-g_{1\max}\leq 0,$$

where $g_{1\max}=1/\mu \ln(mes(\Omega_{3}))$ which is obtained for $\sigma_{12}=\bar{\sigma }$ in all points of space Ω_3_.

The second most commonly used aggregation function for local constraints is the *p* norm approximation governed by the following formula:

(9) $$g_{2}\left(r\right)={\left[{\iint_{\Omega_{3}}\left(\frac{\sigma_{12}-\bar{\sigma }}{\bar{\sigma }}\right)}^{p}d\Omega \right]}^{1/p},$$

which tends to the norm (6) for p→∞. The numerical test using aggregation functions (7) and (9) showed that the application of function (9) ensures faster smoothing of shear stresses. That is why in our further study, we use function (9).

In order to optimize the solder layer topology, we divide space Ω into finite elements. Projection variable r(x) is defined only in space Ω_3_ and is coupled with Young modulus  E(x) and with β(x)=E(x)α(x) as well as with the volume material density ρ(x) of each element from Ω_3_ under the following relations (RAMP scheme [47]):

(10) $$\begin{array}{l} E\left(x\right)=\frac{E_{0}\;r\left(x\right)}{\left(1+a\cdot (1-r\left(x\right)\right)},\;\;\;\;\;\beta \left(x\right)=\frac{\alpha_{0}\;E_{0}\;r\left(x\right)}{\left(1+b\cdot (1-r\left(x\right)\right)},\;\;\;\\ \;\rho (x)=\frac{\rho_{0}\;r\left(x\right)}{\left(1+a\cdot (1-r\left(x\right)\right)},\;\;\;x\in \Omega_{3}, \end{array}$$

where *a*, *b* are the penalty parameters used for the security of compact material distribution, which are selected as a result of a computational experiment, r(x) is the field of design variables $0<r_{0}\leq r\left(x\right)\leq 1$, and $r_{0}$ is a small number which guarantees non-zero stiffness of the finite elements. For r(x)=1 the whole volume is fully filled by the solder material. 

The problem of topological optimization for the task of decreasing the peak values of stresses $\sigma_{12}$ in the solder layer can be formulated in the following way:

(11) $$\underset{r_{0}\leq r\left(x\right)\leq 1}{\min}{\left[{\iint_{\Omega_{3}}\left(\frac{\left|\sigma_{12}\right|}{\bar{\sigma }}\right)}^{p}d\Omega \right]}^{1/p},$$

under the constraint

(12) $$\sigma_{ij,\;j}=0\;\mathrm{in}\;\Omega ,\;\left(i,\;j\;=1,2\right),$$

and subjected to two isoparametric constraints put on the physical density ρ(x):

(13) $$\int_{\Omega_{3}}\rho (x)d\Omega \geq \gamma \cdot mes\left(\Omega_{3}\right)\;,$$

where γ denotes the proportion of the solder material used to build the structure and $mes\left(\Omega_{3}\right)$ is the area of the solder space. 

In order to project the field of design variables into space 0/1, when the boundaries of optimal structure should be precisely defined, a Heaviside filter (HF) is used [48]. HF is a Heaviside step function which projects the field of design variables (now called the transitional field of design variables and denoted by ξ(x)) into the real field of design variables r(x). To simplify the computation of two gradients when conducting a sensitivity analysis to find a solution to the optimization problem, we use the smoothened Heaviside function of the following form:

(14) $$r(x)=1-e^{-k\xi (x)}+\xi (x)e^{-k},$$

which is continuous. Parameter k≥0 stands for the curvature of projection which is linear for k=0, and tends to the unit Heaviside step function for k→∞.

Further, in order to remove the effect of “chess plane”, we define a target function in the approximation process in the form of a linear combination of function (11) and additionally introduced penalty function of the following form:

(15) $$\underset{r_{0}\leq r\left(x\right)\leq 1}{\min}\;\left\{\left(1-q\right){\left[{\iint_{\Omega_{3}}\left(\frac{\left|\sigma_{12}\right|}{\bar{\sigma }}\right)}^{p}d\Omega \right]}^{1/p}+q\frac{h_{0}h_{\max}}{mes\left(\Omega_{3}\right)}\int_{\Omega_{3}}{\left|\nabla \rho \left(x\right)\right|}^{2}d\Omega \right\}.$$

The second term stands for the penalty function, $h_{0}$ is the initial size of the mesh of finite elements and $h_{\max}$ stands for the running mesh size. The quantity 0≤q≤1 (given coefficient) makes it possible to balance the target function and the penalty function. A solution to the so far defined optimization problem has been presented using the method of moving asymptotes [51]. We have used FEM with linear triangle FE and non-uniform mesh compressed in the vicinity of the adhesion layer. Finite element calculations have been performed using Comsol Multiphysics v. 5.3.0.316. An analysis of the problem solutions for a different number of finite elements has been carried out, the refinement of the partition grid does not lead to a significant improvement in the result, which indicates the results stability. The used number of FEs is 14000 with 1640 nodes. The number of degrees of freedom is 3280.

## 4. Numerical Results of the Topological Optimization of Micro-Electronic Packages under Thermal Load

### 4.1. Case Study 1

**Figure 2:**
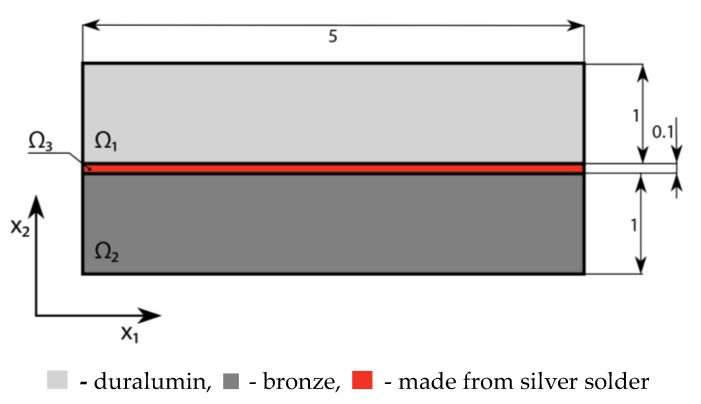
Design of a symmetric three-layer package (Unit: mm).

We consider the thermoelastic three-layer package without mechanical loads (see Figure 2). The package is heated up to the temperature *θ* = 100 °C over the whole volume. Space Ω_1_ is made from duralumin with Young’s moduli *E*_1_ = 70.56 × 10^9^ Pa and *α*_1_ = 23.8 × 10^−6^ K^−1^. The material of space Ω_2_ is bronze with *E*_2_ = 110 × 10^9^ Pa and *α*_2_ = 17.5 × 10^−6^ K^−1^. Solder joint space Ω_3_ is made from silver with *E*_3_ = 83 × 10^9^ Pa and *α*_3_ = 19.5 × 10^−6^ K^−1^ (see the package size in millimeters presented in Figure 2).

While solving the problem of optimization within the scheme of approximation of material based on (10), the following coefficients are fixed: *a* = 3 and *b* = 5. In the target function (15), we used *p* = 7 and *q* = 0.25. The values of these parameters were obtained as a result of numerical experiments for the problems under consideration.

**Figure 3:**
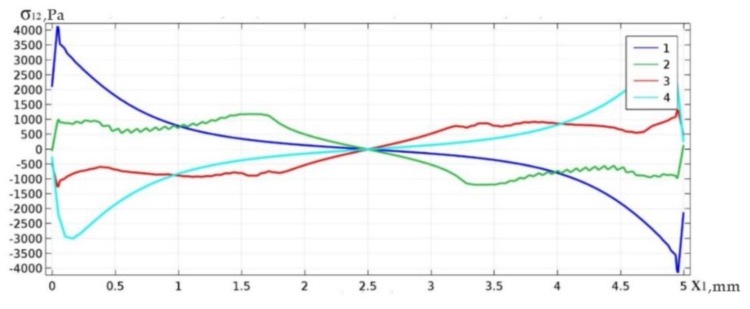
Curves of shear stress distribution on the upper (1,2) and bottom (3,4) boundary of the solder joint area.

Figure 3 shows examples of the distribution of shear stresses along the upper and lower boundary of space Ω_3_ for the case of fully filled solder material (curves 1 and 4) and for the case of topologically optimal structure for *γ* = 0.6 (curves 2 and 3). In Figure 3, and later in the paper, the vertical axis shows the values of shear stresses *σ*_12_*Pa*, whereas the horizontal axis shows the coordinate *x*_1_[*mm*] along the solder layer. 

From the distribution of curves, it can be concluded that in the case study under consideration, the optimization process implied a decrease of the peak values of shear stresses by more than 3 (4) times for the upper (lower) solder boundary. 

**Figure 4:**
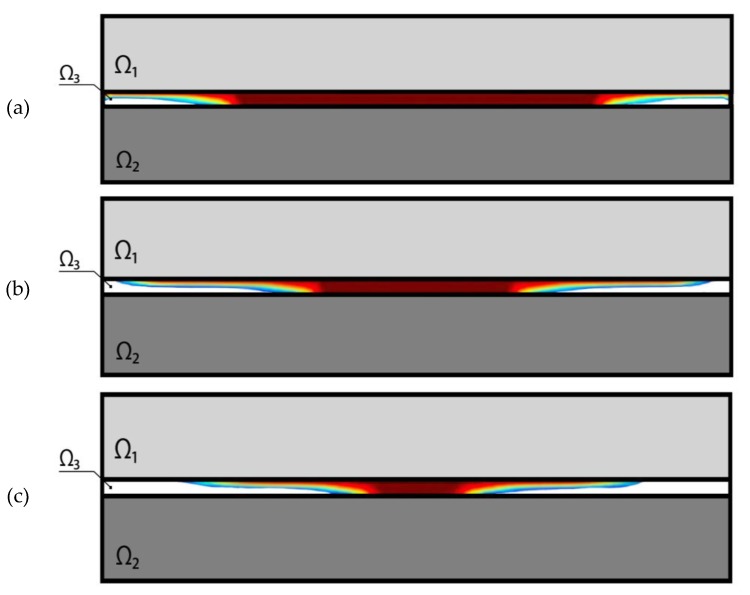

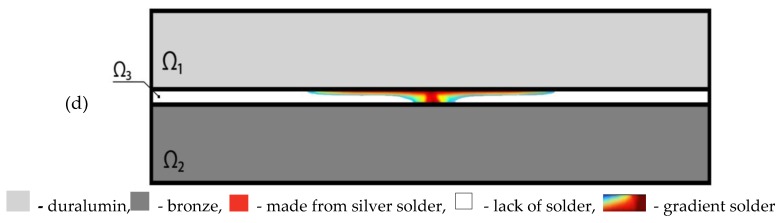
Topology of the optimal solder structure for symmetric three-layer package. (**a**) *γ* = 0.8; (**b**) *γ* = 0.6; (**c**) *γ* = 0.4; (**d**) *γ* = 0.2.

Table 1 gives references to the figures on which the optimal distribution of the solder structure along the area Ω_3_ for different values of *γ*, i.e., the amount of the solver solder is obtained. In Figure 4, and Figures 6, 8, 10, areas filled with solder with a Young’s modulus value of *E*_3_ are indicated in red, and voids are indicated in white. The transition colors correspond to the gradient properties of the solder with the Young’s modulus varying from 0 to *E*_3_. The third column of the table gives the averaged value of shear stress along the solder joint area

(16) $$\bar{S}=\frac{1}{mes\left(\Omega_{3}\right)}\iint_{\Omega_{3}}\sigma_{12}d\Omega .$$

The percentage decrease in the peak values of shear stress in the optimal structure with respect to the peak values of shear stresses $\Delta =\frac{\sigma_{12}^{Heonm}-\sigma_{12}^{onm}}{\sigma_{12}^{Heonm}}\cdot 100\%$ of the input non-optimal construction is presented in the fourth column.

Based on the results shown in Table 1, the following conclusions were drawn. The smallest value of the averaged tangential stresses $\bar{S}$ in the solder layer is obtained for the minimum value of the solder amount *γ*. It is in agreement with physical expectations, i.e., the contact area of the first and third layer is reduced, and consequently the difference of their heating properties plays a less important role. In all cases the gradient of the optimal solder construction is exhibited, i.e., its non-homogeneity along both thickness and length.

### 4.2. Case Study 2

**Figure 5:**
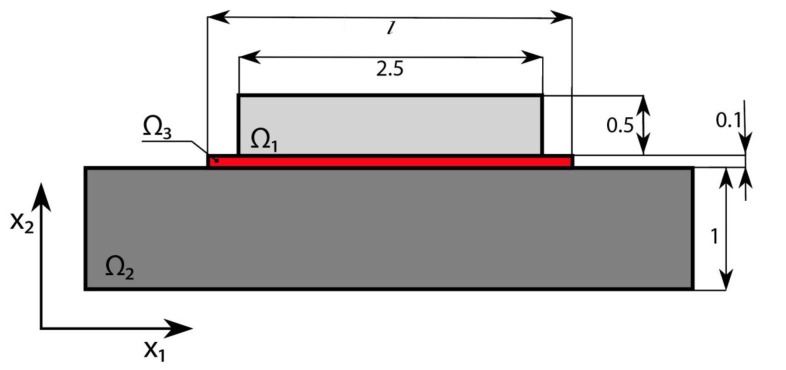
Construction of the non-symmetric package (Unit: mm).

We consider a thermoelastic three-layer package free from external mechanical loads shown in Figure 5. In contrast to the previous example, areas Ω_1_ and Ω_2_ have different geometric dimensions as well as different sizes and forms of the solder area Ω_3_. Other parameters and material characteristics are selected in the same way as in case study 1.

**Figure 6:**
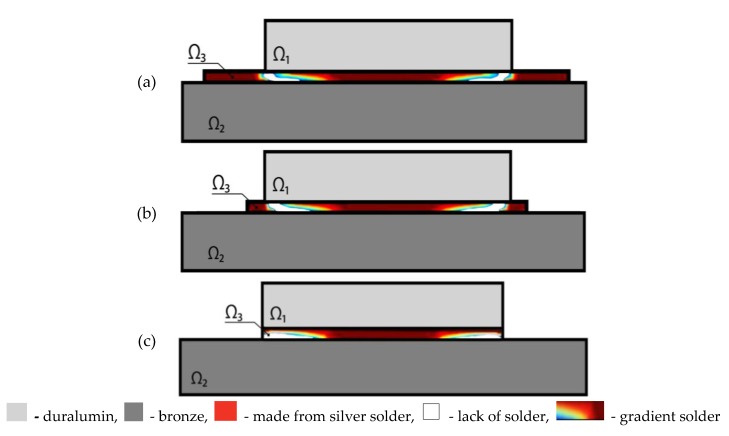
Topology of the optimal structure for the solder of the non-symmetric package. (**a**) *l =* 4, *γ* = 0.75; (**b**) *l =* 3, *γ =* 0.75; (**c**) *l =* 2.5, *γ =* 0.75.

We investigated the topologically optimal microstructure of the solder with respect to length *l* of layer Ω_3_ (it is shown in the first column of Table 2). The second column presents the form of area Ω_3_ with the optimal structure of the solder material distribution (Figure 6).

**Figure 7:**
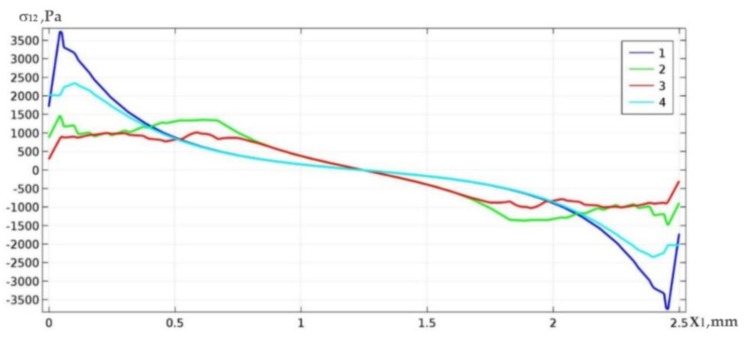
Curves of the distribution of shear stresses along the upper (1,2) and bottom (3,4) boundary of the solder area.

The allowed values of the solder amount are shown in the third column of the Table 2: value *γ* = 1 corresponds to the initial non-optimal construction with fully filled solder area Ω_3_ (*γ* = 0.75). In the fourth column, the average value of shear stress $\bar{S}$ follows from formula (14). In the fifth column, the percentage decrease of the peak shear stresses in the optimal structure with respect to the peak value of counterpart stresses in the input non-optimal construction is given (see Figure 7).

As can be seen from Table 2, the introduction of additional areas of the solder by extending the length of the layer does not give the expected additional decrease in the peak value of shear stresses. The fundamental solder structure in all considered cases can be found between the upper and bottom package layers while the remaining zones of the solder do not affect the level of tangential stresses and can be neglected. Therefore, the most optimal microstructure is the one which along the length of the solder layer is equal to length of the upper layer *l* = 2.5 mm.

Figure 7 presents distributions of shear stresses of the non-optimal (curves 1, 4) and optimal (curves 2, 3) solder structure with respect to the upper and bottom layer boundary Ω_3_ for *l* = 2.5 mm. As can be seen in Figure 7, as a result of the optimization, a double decrease in the peak shear stresses at the upper and bottom solder boundaries was achieved.

**Figure 8:**
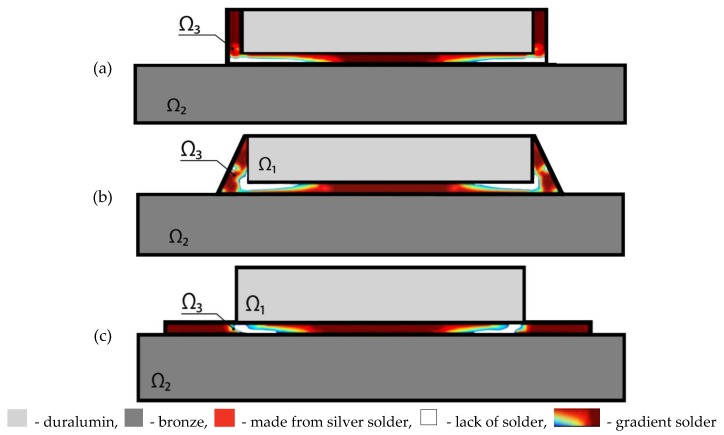
Topology of the solder structure. (**a**) the solder is located around the structure; (**b**) the solder is arranged in a pyramid shape; (**c**) the solder is located under the structure.

In addition to the solder in the form of a straight layer Ω_3_, we consider other possible geometric forms of the solder area. The first column of Table 3 gives data in which the input forms of area Ω_3_ are compared with optimal gradients of the microstructures of the solder distribution (Figure 8). The second column of Table 3 shows the given area of space Ω_3_. The third column shows the values of the allowed amount of solder *γ*. The fourth column presents the values of shear stresses $\bar{S}$ computed using formula (16). The fifth column gives the percentage decrease in the peak values of shear stresses in the optimal microstructure with respect to the peak values of counterpart shear stresses in a non-optimal construction.

As can be seen from Table 3, the introduction of additional solder parts located outside the domain between the package layers did not result in an additional reduction of peak shear stresses. The fundamental solder structure is located between upper and bottom layers of the package, and the remaining zones of the solder do not play any important role and can be neglected. Therefore, the most optimal structure is the one which occurs in the third row of Table 3.

## 5. Topological Optimization of Non-Symmetric Packages under Thermal and Mechanical Loads: Numerical Results

The packages can be connected using soldering in different ways: by using a continuous solder, flange bevel solder, square groove solder, double butt solder, arc solder, fillet solder, etc. In the solder joints, the Stresses are distributed non-uniformly. Stress peaks occur in the points of geometric discontinuities. In general, the linkage by solder involves the occurrence of discontinuities at the ends of the solder. The occurring non-uniformities generate bending moments originating from eccentric loads and caused by non-uniform distribution of moments around the solder layer. These moments imply the occurrence of disruptive normal stresses in the solder layer. There are ways to reduce the harmful effects of the emerging eccentric loads in the solder layers. For instance, it is known that it is useful to apply the narrowing of edges of solder layers and introduce pre-bending. The decrease of the maximum shear stresses can be achieved by increasing the length and thickness of solder, the thickness of the combined layers and reducing the solder modulus. In the given example, all geometric parameters of the linkage remain constant, while a decrease in the maximum values of stresses at the ends of the solder is achieved by topological optimization of the solder structure.

**Figure 9:**
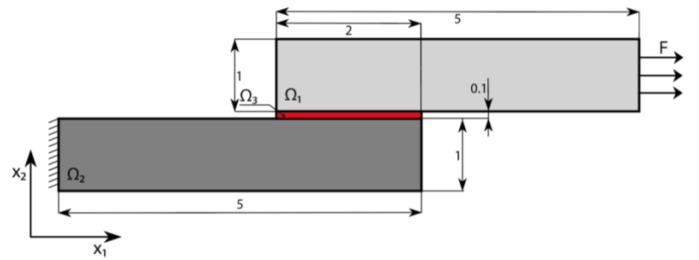
Construction of the package and boundary conditions (Unit: mm).

**Figure 10:**
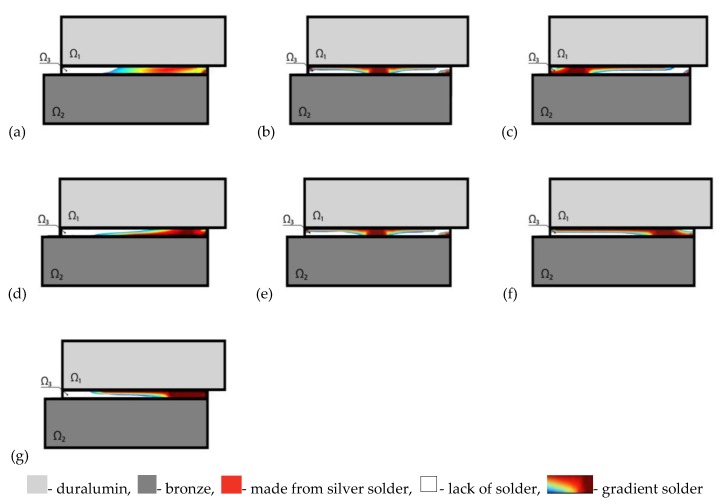
Topology of the optimal solder construction under thermal and mechanical loads. (**a**) *θ* = 0 °C, F = 3000 N; (**b**) *θ* = 100 °C, F = 0 N; (**c**) *θ* = 100 °C, F = 1000 N; (**d**) *θ* = 100 °C, F = 3000 N; (**e**) *θ* = −100 °C, F = 0 N; (**f**) *θ* = −100 °C, F = 1000 N; (**g**) *θ* = −100 °C, F = 3000 N.

Let us consider the construction shown in Figure 9. In contrast to the previous cases, despite thermal excitation, the mechanical load along the *x*_1_ axis is introduced. The non-homogenous distribution of the moments around the welding layer results in the appearance of additional shear thermal stresses in the welding joint.

The obtained results are included in Table 4. The first column presents input temperature *θ*[°C] The second column contains mechanical load *F*[*N*]. The third column refers to the figures, where the optimal weld microstructure distribution in space Ω_3_ is obtained. The average values of shear stresses $\bar{S}$ are shown in the fifth column, while the sixth column gives the decrease in peak values of shear stresses (in percent) in the optimal microstructure with respect to the peak values of the counterpart shear stresses in the input non-optimal construction.

**Figure 11:**
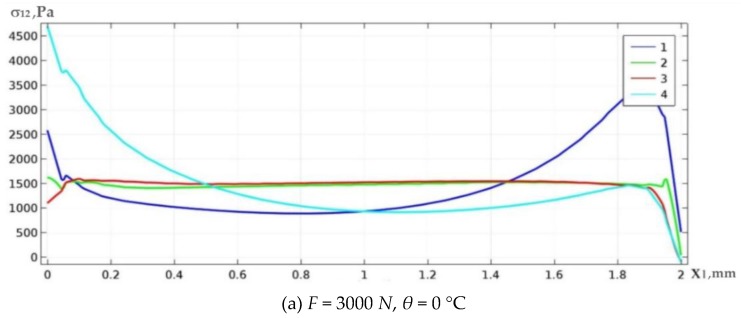

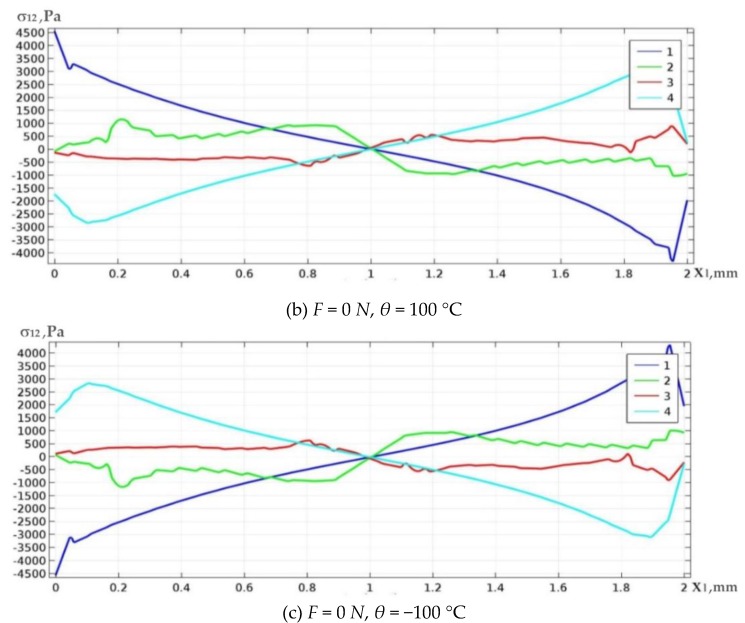
Graphs of the shear stress distribution along upper (1, 2) and bottom (3, 4) boundary of the solder layer under mechanical (**a**) and thermal (**b**,**c**) load.

Below, we present graphs of shear stresses in various cases of the mentioned loads: only the mechanical load is applied (Figure 11a) and only thermal load is applied (Figure 11b,c). Curves 2 and 3 of stress values *σ*_12_ in all figures correspond to optimal constructions, while curves 1 and 4 correspond to the input (non-optimized) constructions. We begin with the optimization process of the weld microstructure subjected only to the mechanical load. In a given case, the averaged value $\bar{S}$ for both the optimized and input microstructure of the welding layer remains unchanged, which requires balancing of extension load *F*. However, the peak stress values are reduced by more than 4.5 (1.5) times in the lower (upper) boundary.

The action of thermal load only (*F* = 0 *N*, *θ* = 100 °C, Figure 11b, and *F* = 0 *N*, *θ* = −100 °C, Figure 11c) implies a decrease of both $\bar{S}$ and peak values *σ*_12_ by 4 (4.5) times on the upper (lower) boundary of the solder layer for *θ* = 100 °C and by 4.5 (4) times on the upper (lower) boundary of the solder layer for *θ* = −100 °C. 

**Figure 12:**
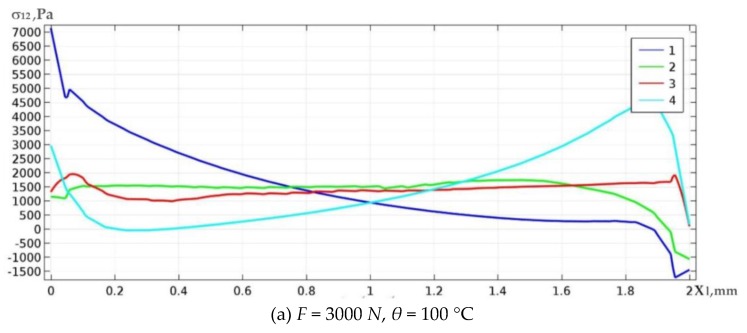

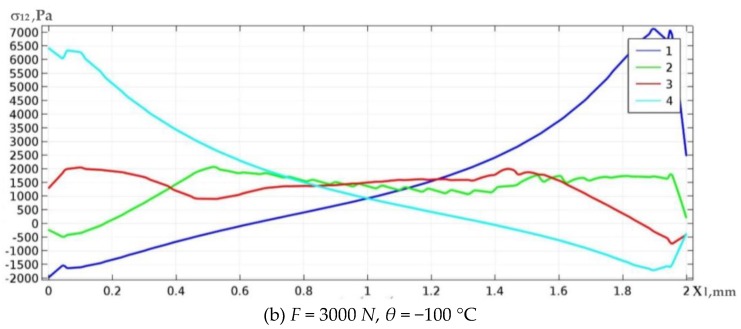
Graphs of the distribution of shear stresses along the upper (1, 2) and bottom (3, 4) boundary of the solder area under simultaneous action of both mechanical (positive (**a**) and negative (**b**) thermal loads.

Simultaneous action of both mechanical and thermal load for *F* = 3000 N, *θ* = 100 °C (Figure 12a) on the upper (lower) boundary of the solder welding layer reduces the peak shear stresses $\sigma_{12}$ by 3.7 (3) times. In the case of *F* = 3000 N, *θ* = −100 °C (Figure 12b) the counterpart values for the upper (lower) boundary correspond to the decrease of peak stresses by 4 (3) times. 

This work continues a series of publications by authors dedicated to topological optimization [52,53,54].

## 6. Concluding Remarks

Based on the investigations carried out, the following conclusions can be formulated.

1. A novel topological optimization methodology of structural soldered joints is developed.
2. The proposed methodology yielded stresses decrease for an optimal structure.
3. The main advantage of using topological optimization is that when designing structural elements, you do not need to know their geometry in advance.
4. The numerical implementation of the developed methodology for strain calculations is performed by the finite element method using the method of moving asymptotes.
5. In all cases, the constructed optimized structures demonstrate a significant decrease in the stress peaks in the solder layer at a smooth distribution of stresses along it, compared with a uniform solder layer.
6. The reliability is confirmed by the coincidence of numerical solutions obtained on the basis of the developed methodology for topological optimization with experimental data [6,7,8]. Peak stresses occur in small areas of the solder layer located near the connection ends; this coincides with the experimental results not reported here.
7. The developed methodology based on topological optimization can be used in the design of macro and micro structures.

## Figures and Tables

**Table 1:** Results of the topological optimization for the symmetric three-layer package.

*γ*	Topology of the Optimal Solder Structure	$\bar{S}$ [GPa]	Δ [%]
1	Original construction	1755.34	0.0
0.8	Figure 4a	894.74	60.0
0.6	Figure 4b	820.41	70.0
0.4	Figure 4c	700.14	75.0
0.2	Figure 4d	410.52	87.5

**Table 2:** Results of topological optimization for the non-symmetric package.

*l*	Topology of Optimal Solder Structure	*γ*	$\bar{S}$ [GPa]	Δ [%]
4	Original construction	1	603.95	0.0
	Figure 6a	0.75	564.93	60.0
3	Original construction	1	804,41	0.0
	Figure 6b	0.75	748.16	60.0
2.5	Original construction	1	953.92	0.0
	Figure 6c	0.75	876.61	61.0

**Table 3:** Optimal graded microstructures of the solder distribution.

Topology of Optimal Solder Structure	*mes*(Ω_3_)	*γ*	$\bar{S}$ [GPa]	Δ [%]
Figure 8a	0.4	1	771.65	0.0
		0.75	656.80	50.0
Figure 8b	0.4	1	809.65	0.0
		0.75	739.75	60.0
Figure 8c	0.4	1	603.98	0.0
		0.75	564.92	60.0

**Table 4:** Comparing results of the topological optimization under thermal and mechanical loads with the original construction.

θ	*F* [N]	Topology of Optimal Solder Structure Shown in Figures	*γ*	$\bar{S}$ [GPa]	Δ [%]
	3000	Original construction	1	1501.15	0.0
		Figure 10a	0.5	1501.00	78
100	0	Original construction	1	1510.10	0.0
		Figure 10b	0.5	620.00	72
	1000	Original construction	1	1400.90	0.0
		Figure 10c	0.5	698.90	77.3
	3000	Original construction	1	1524.95	0.0
		Figure 10d	0.5	1508.7	71.5
−100	0	Original construction	1	1510.10	0.0
		Figure 10e	0.5	620.00	72
	1000	Original construction	1	1661.95	0.0
		Figure 10f	0.5	760.55	75
	3000	Original construction	1	2080.00	0.0
		Figure 10g	0.5	1562.90	69
